# Targeting Microglia to Treat Degenerative Eye Diseases

**DOI:** 10.3389/fimmu.2022.843558

**Published:** 2022-02-17

**Authors:** Sean K. Wang, Constance L. Cepko

**Affiliations:** ^1^ Department of Ophthalmology, Byers Eye Institute, Stanford University School of Medicine, Palo Alto, CA, United States; ^2^ Department of Genetics, Blavatnik Institute, Harvard Medical School, Boston, MA, United States; ^3^ Department of Ophthalmology, Harvard Medical School, Boston, MA, United States; ^4^ Howard Hughes Medical Institute, Chevy Chase, MD, United States

**Keywords:** microglia, retina, neurodegeneration, neuroinflammation, neuroprotection, immunomodulation, therapies

## Abstract

Microglia have been implicated in many degenerative eye disorders, including retinitis pigmentosa, age-related macular degeneration, glaucoma, diabetic retinopathy, uveitis, and retinal detachment. While the exact roles of microglia in these conditions are still being discovered, evidence from animal models suggests that they can modulate the course of disease. In this review, we highlight current strategies to target microglia in the eye and their potential as treatments for both rare and common ocular disorders. These approaches include depleting microglia with chemicals or radiation, reprogramming microglia using homeostatic signals or other small molecules, and inhibiting the downstream effects of microglia such as by blocking cytokine activity or phagocytosis. Finally, we describe areas of future research needed to fully exploit the therapeutic value of microglia in eye diseases.

## Introduction

First described by Pío del Río-Hortega in the early 20^th^ century, microglia are the resident immune cells of the central nervous system (CNS), including the retina. In healthy eyes, microglia comprise approximately 0.3-1.0% of retinal cells and perform a multitude of functions, such as immune surveillance, synaptic refinement, neurotrophic support, and clearance of debris (1–4). Under homeostatic conditions, retinal microglia primarily reside in the inner and outer plexiform layers and possess a ramified morphology with long motile processes that dynamically monitor the ocular environment (1, 2). However, in response to local injury, infection, or broader insults like hypoxia, microglia can transition from their normal quiescent state to activated phenotypes hallmarked by increased cytokine secretion and phagocytic activity (5–7).

**Figure 1:**
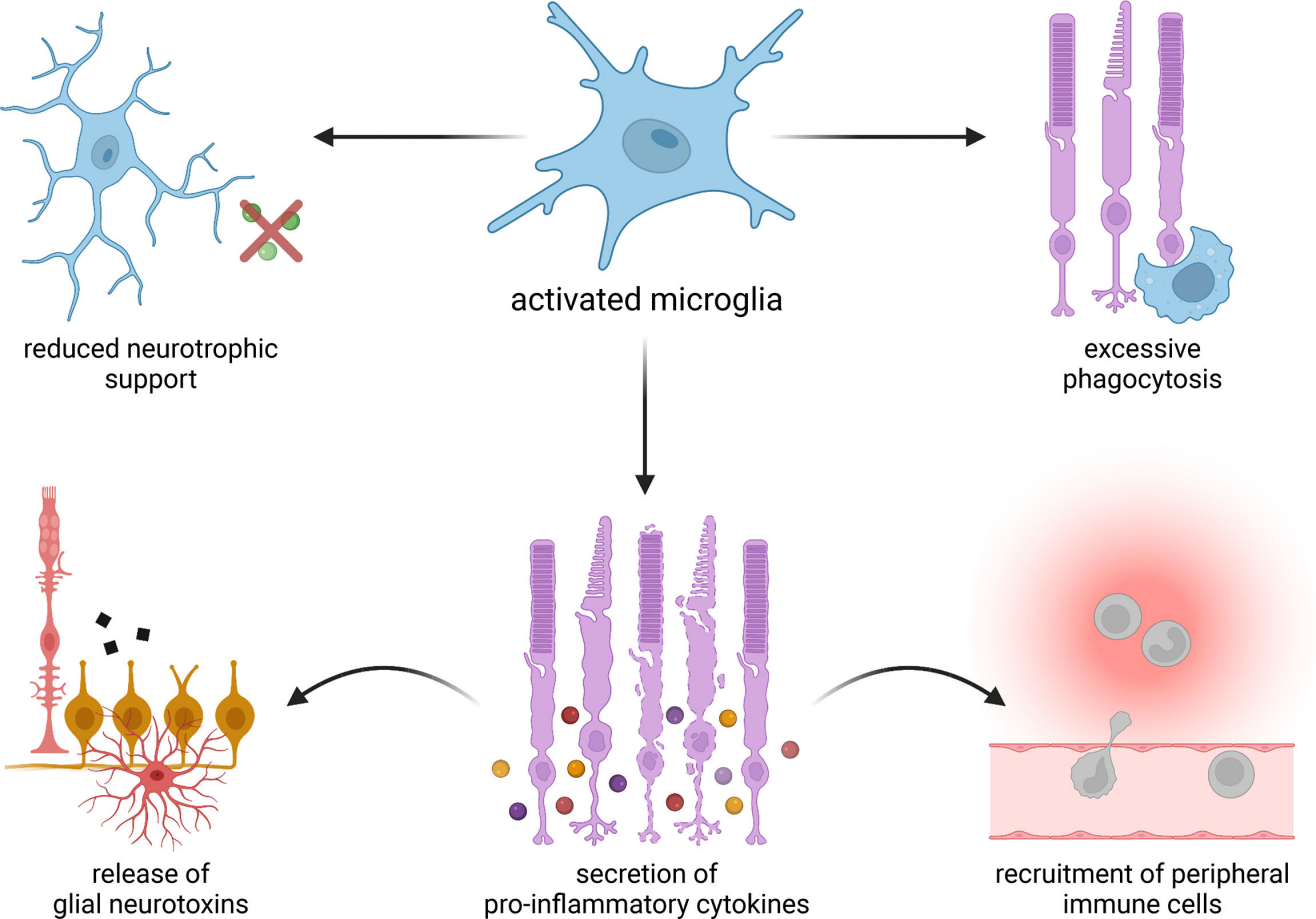
Proposed mechanisms of microglia-mediated damage in degenerative eye diseases. Activated microglia may provide less robust neurotrophic support and carry out excessive phagocytosis. They may also secrete pro-inflammatory cytokines, which in turn can induce the release of neurotoxins from resident glial cells as well as recruit immune cells from outside of the eye.

Activation of microglia has been observed in virtually every major neurodegenerative disorder (8, 9), as well as numerous degenerative and inflammatory diseases of the eye (10–13). Indeed, microglia have been implicated in the progression of retinitis pigmentosa (RP), age-related macular degeneration (AMD), glaucoma, diabetic retinopathy (DR), uveitis, and retinal detachment among other ocular conditions (14–19) (**Figure 1**). While these diseases differ in their underlying etiologies, they are all characterized by the loss of photoreceptors or retinal ganglion cells (RGCs), resulting in deterioration of vision and, in some cases, blindness. It is possible that interventions targeting microglia could alleviate photoreceptor and RGC death, thereby helping patients preserve their sight.

At the same time, there is a growing appreciation that microglia can also take on beneficial roles during eye disease. In a handful of animal models, improved tools to experimentally remove microglia have unexpectedly revealed these cells to be neutral or even protective against degeneration (19–24). Microglia may further exhibit distinct functions depending on the stage and chronicity of the disorder, and microglial populations at different physical locations in the eye might behave differently. These complexities highlight the need for equally nuanced treatment approaches to fully leverage the therapeutic value of microglia.

**Figure 2:**
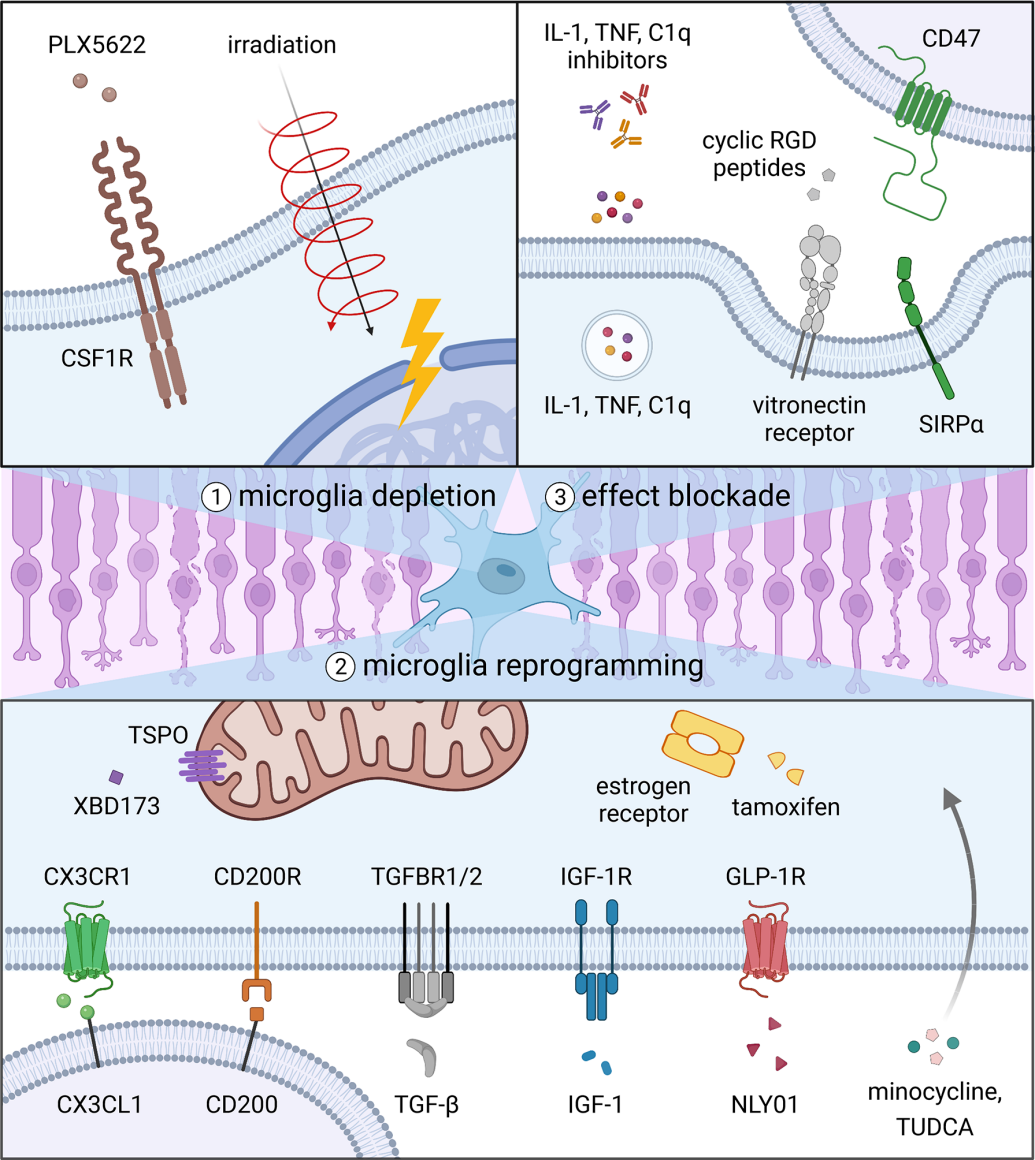
Strategies to target microglia in degenerative eye diseases. Current options include 1) depleting microglia with small molecules or radiation, 2) reprogramming microglia with signals that modify microglial activation, and 3) blocking the downstream effects of microglia by inhibiting cytokine activity or phagocytosis.

In this review, we discuss strategies that could be used to therapeutically target microglia and their potential to ameliorate degenerative diseases of the eye. These include 1) depleting microglia with small molecules or radiation, 2) reprogramming microglia with signals that modify microglial activation, and 3) blocking the downstream effects of microglia by inhibiting cytokine activity or phagocytosis (**Figure 2**). For each specific approach, we evaluate data from pertinent animal models and clinical trials, if available, to assess how the intervention might perform in patients with different ocular conditions (**Table 1**). Lastly, we propose several avenues of future research on microglia that may lead to better therapies for eye disorders.

**Table 1:** Interventions targeting microglia supported by in vivo loss- or gain-of-function experiments.

Intervention	RP	AMD	Glaucoma	DR	Uveitis	RD
Depletion						
CSF1R inhibitors	o	+	o		+	–
Irradiation	+		+			
Reprogramming						
Minocycline	+		+	+	+	+
CX3CL1	+	+	+	+	+	
CD200	o	+			+	
TGF-β	+	–				
TSPO ligands	o	+				
IGF-1	+		+			
TUDCA	+	+		+		+
Tamoxifen	+					
GLP-1R agonists			+	+		
Effect blockade						
IL-1 inhibitors	+	+		o	+	+
TNF inhibitors	+	+	+	+	+	+
C1q inhibitors	–	o	+		o	
Cyclic RGD peptides	+	+				
CD47	+					

Interventions with available data are denoted as beneficial (+), detrimental (–), or neutral (o).

RP, retinitis pigmentosa; AMD, age-related macular degeneration; DR, diabetic retinopathy; RD, retinal detachment; CSF1R, colony-stimulating factor 1 receptor; TGF-β, transforming growth factor beta; TSPO, translocator protein; IGF-1, insulin-like growth factor 1; TUDCA, tauroursodeoxycholic acid; GLP-1R, glucagon-like peptide 1 receptor; IL-1, interleukin 1; TNF, tumor necrosis factor; C1q, complement component 1q; RGD, Arg-Gly-Asp.

## Strategies to Target Microglia in Eye Disease

### Depletion

Much of what we know about microglial function comes from studies in which these cells were depleted. Early efforts to remove microglia were made possible by the creation of CD11b-HSVTK transgenic mice, which express the herpes simplex virus-derived thymidine kinase (HSVTK) suicide gene in CD11b-positive cells, including microglia (25). However, it was not until the identification of CX3CR1 as a marker for microglia and the generation of CX3CR1^CreER^-DTR mice harboring the diphtheria toxin receptor (DTR) gene that selective ablation of microglia could be achieved (26, 27). While these transgenic methods have enabled efficient elimination of microglia in mice, genetic targeting of microglia in humans is not currently possible. Alternative strategies for depleting microglia that could also be used in patients include pharmacologic inhibitors and irradiation, which are detailed below.

#### CSF1R Inhibitors

The most widely used method to deplete microglia in experimental settings is inhibition of colony-stimulating factor 1 receptor (CSF1R), a membrane protein expressed by microglia that is essential for their survival (28). This approach has largely replaced other compounds used to remove macrophages, such as clodronate liposomes, which have difficulty crossing the blood-retina barrier and are less specific for microglia due to effects on peripheral immune cells (23). When administered to mice, small molecule inhibitors of CSF1R such as PLX3397 (pexidartinib) and PLX5622 can eliminate up to 100% of retinal microglia in one to two weeks (18, 20, 21, 29). Pexidartinib furthermore has an established safety profile in humans, as it was recently approved for the treatment of tenosynovial giant cell tumors (30).

In several animal models, depletion of microglia with CSF1R inhibitors has been shown to counter the progression of ocular pathology. For instance, in mice treated with laser to induce choroidal neovascularization (CNV), a hallmark of exudative AMD, administration of PLX5622 led to significantly faster resolution of lesions (29). Microglial ablation in mice with PLX5622 also suppressed the development of experimental autoimmune uveoretinitis (EAU), a model of human autoimmune uveitis, although the outcome depended on the timing of disease (18). Specifically, suppression was only observed if PLX5622 was given during the early stages of EAU, suggesting that microglia are essential for the initiation of ocular autoimmunity but subsequently play a more limited role.

Conversely, there are multiple models of eye disorders in which CSF1R inhibitors have failed to demonstrate a therapeutic benefit. In *rd10* mice, which carry a mutation that causes autosomal recessive RP (31), early genetic ablation of microglia using a diphtheria toxin system improved the survival of rod photoreceptors (32). However, in *rd1* animals, which harbor a more severe mutation in the same gene, microglia removal using PLX3397 or PLX5622 did not alter the rate of photoreceptor death (20–22). For RGCs, microglia depletion with PLX5622 had no observable effect after optic nerve crush and failed to preserve visual function in mice injected with microbeads to raise intraocular pressure (IOP) (23, 24). When given before retinal detachment in mice, PLX5622 also increased the number of apoptotic photoreceptors (19), indicating that microglia during acute retinal detachment protect against rather than promote degeneration. Collectively, these studies suggest that in certain conditions such as exudative AMD or early uveitis, microglia may in fact be predominantly harmful. Nonetheless, for many eye disorders, simply eliminating activated microglia will not be sufficient to slow the course of the disease.

#### Irradiation

Forms of ionizing radiation such as gamma rays and X-rays are routinely used in clinical settings to perform diagnostic imaging and treat cancers. Coincidentally, exposure to ionizing radiation can also result in microglia depletion. In mice, a single dose of gamma radiation progressively eliminated an average of 75% of retinal microglia after 70 days (33). Loss of microglia was likely from apoptosis secondary to DNA double-strand breaks, as has been observed for brain microglia following similar irradiation (34).

In 2005, Anderson et al. reported that large doses of gamma radiation unexpectedly preserved RGCs in DBA/2J mice, which develop congenital glaucoma due to pigment dispersion (35). After irradiation at five weeks of age, the percentage of damaged optic nerves dramatically improved from 83.3% in untreated 12-month-old animals to only 4.9%. In a follow-up study, the same group found that localized irradiation of DBA/2J eyes using X-rays could specifically protect treated eyes while those contralateral underwent degeneration (36). IOP remained elevated in both eyes and was unaltered by irradiation. However, only irradiated eyes showed reduced expression of IBA1 (36, 37), a marker for microglia, suggesting that depletion of microglia may have contributed to saving RGCs.

Outside of DBA/2J mice, low-dose gamma radiation has also been found to delay photoreceptor death in both the *rd1* and *rd10* models of RP (38). However, photoreceptor preservation in these mice was unique to low-dose radiation, as increasing either the dose rate or total dose as little as four-fold abolished the rescue (38). Since higher doses of radiation eliminate more microglia (39), these data argue that neuroprotection from ionizing radiation is not solely due to microglia depletion. Indeed, while irradiation helped in animal models of RP and glaucoma, treatment with CSF1R inhibitors, which more efficiently remove microglia, did not (20–22, 24). Because microglia depletion after irradiation is relatively slow and incomplete, some of its therapeutic effects might come from changes induced in the microglia that survive. Alternatively, the benefits of irradiation could be due to concomitant elimination of other cell types in the eye, such as infiltrating monocytes (36). Regardless, any possible benefits of irradiation as an ocular therapy should be balanced against the risk for radiation retinopathy, a complication of radiation exposure characterized by capillary occlusions and microaneurysms (40).

### Reprogramming

Despite its potential to alleviate pathology in several eye disorders, microglia depletion is not without consequences. Long-term ablation of microglia results in failure to maintain the synapses used by photoreceptors, leading to impaired retinal signaling (41). Furthermore, microglia in some conditions appear to perform favorable actions, since elimination of these cells has a neutral or even detrimental effect on disease progression (19–24). For these reasons, reprogramming rather than removing microglia may be an appealing therapeutic approach to correct microglial dysregulation while preserving or enhancing their beneficial functions.

Below, we review a number of candidate therapies that attempt to achieve microglia reprogramming, defined here as the redirection of microglia away from harmful phenotypes. These interventions range from small molecules and repurposed drugs to proteins involved in the endogenous regulation of microglia. While not comprehensive, this list reflects the diversity of molecular pathways implicated in microglial activation and how they might be co-opted to treat degenerative eye diseases.

#### Minocycline

Minocycline is a tetracycline-derived antibiotic that readily crosses the blood-retina barrier and possesses anti-inflammatory properties independent of its anti-microbial activity. As early as 1998, minocycline was found to inhibit pro-inflammatory cytokine production by microglia in a model of brain ischemia (42), leading to evaluation of the compound as a potential therapeutic for multiple CNS pathologies (43–45). Mechanistically, minocycline is thought to inhibit the pro-inflammatory “M1” polarization of microglia as evidenced by its attenuation of markers including interleukin 1 beta (IL-1β) and tumor necrosis factor (TNF) (46). In contrast, expression of anti-inflammatory “M2” genes such as IL-4 and IL-10 are largely unaffected by minocycline and likely dominate following the drug’s suppression of M1 polarization (46).

Across animal models of degenerative eye disease, treatment with minocycline has often demonstrated promise. In the *rd10* model of RP, twice daily systemic injections of minocycline reduced rod death and partially preserved retinal function as measured by both scotopic electroretinography (ERG) and a visual behavior test (47). These changes coincided with greater ramified morphology among retinal microglia and decreased immunoreactivity for the lysosomal marker CD68, in addition to lower levels of TNF protein in the retina (47, 48). For glaucoma, minocycline improved RGC survival in mice with transiently elevated IOP as well as in the DBA/2J model of chronic glaucoma secondary to pigment dispersion (49, 50). In the latter, the proportion of microglia with ramified morphology was also increased (50), supporting the notion that microglial deactivation by minocycline preserved RGCs.

In rats made diabetic by streptozotocin (STZ), a compound which destroys pancreatic β-cells, the activity of caspase-3 in the retina was seen to rise due to increased apoptosis (51). Twice daily treatment with minocycline in these animals restored retinal caspase-3 activity close to non-diabetic levels (51), suggesting that minocycline might lower apoptosis in DR. In a rat model of uveitis induced by intravitreal lipopolysaccharide (LPS), minocycline was also found to be beneficial (52). Specifically, minocycline reduced the number of anterior chamber cells and attenuated RNA expression of IL-1β, IL-6, CCL2, and TNF in the retina, although all these values remained elevated compared to normal eyes (52). Finally, minocycline alleviated photoreceptor loss after induction of retinal detachment in mice, even if given 24 hours after the detachment (53). Minocycline may thus be applicable as an initial treatment option for patients who experienced a retinal detachment and are awaiting definitive surgical repair.

Positive preclinical data for minocycline have since sparked several clinical trials to test the drug in eye diseases. In a pilot phase I/II study enrolling five patients with mild to moderate DR associated with macular edema, twice daily oral minocycline for six months resulted in a small improvement in visual acuity (NCT01120899) (54). However, the trial did not include a control group for comparison, and overall, the effect size was modest. Other ongoing trials involving minocycline include its use in RP (NCT04068207), geographic atrophy associated with AMD (NCT02564978), and branch retinal vein occlusion (NCT01468831).

#### CX3CL1

Microglial homeostasis is maintained by a number of signaling molecules that bind to receptors on the surface of microglia to suppress their activation (55). The best characterized of these signals is CX3CL1, also known as fractalkine, which serves as the sole ligand for the CX3CR1 receptor (56). Constitutively produced by neurons, CX3CL1 is a transmembrane protein whose extracellular domain can undergo proteolytic cleavage to release soluble CX3CL1 (57). Conversely, CX3CR1 expression in the eye is restricted to microglia (20), although the receptor can additionally be seen on several immune populations such as monocytes, natural killer cells, and dendritic cells (26).

In *rd10* mice, removal of CX3CL1-CX3CR1 signaling *via* knockout of CX3CR1 increased microglial phagocytic activity and production of pro-inflammatory cytokines while exacerbating photoreceptor degeneration (58). Microglial activation in *rd10* retinas was also ameliorated after intravitreal injection of recombinant CX3CL1, suggesting a protective role for CX3CL1 in RP (58). Consistent with these findings, overexpression of soluble but not full-length CX3CL1 in the eye improved cone photoreceptor survival in three different mouse models of RP and slowed vision loss (20). However, preservation of cones with soluble CX3CL1 was still observed when microglia were depleted, arguing that a microglia-independent mechanism was at least partially responsible for the rescue effect (20).

In other ocular disease models, absence of CX3CL1-CX3CR1 signaling has similarly been shown to worsen outcomes. Following laser injury to induce CNV, mice with CX3CR1 knocked out exhibited larger lesions than those with intact expression (15). Aged mice lacking CX3CR1 also accumulated microglia in the subretinal space and developed outer retinal thinning and drusen-like deposits reminiscent of those seen in AMD (15). CX3CR1 deficiency in mice furthermore led to more extensive RGC death after transient IOP elevation (49), increased vascular pathology in STZ-induced DR (59), and greater disease severity following induction of EAU (60). Endogenous CX3CL1 signaling thus shields against degeneration in multiple models of eye disorders. An important next step will be to determine if exogenous CX3CL1 in these animals can likewise alleviate cell and vision loss.

#### CD200

CD200 is another broadly expressed transmembrane protein that binds to its receptor, CD200R, on the surface of microglia to inhibit their activation. In the mouse brain and spinal cord, CD200 deficiency leads to less ramified microglia and upregulation of pro-inflammatory markers such as TNF and inducible nitric oxide synthase (iNOS) (61, 62), an enzyme that helps generate reactive nitrogen species. Retinal microglia from CD200 knockout mice similarly show much higher iNOS levels than wild-type controls (63), suggesting that CD200 tonically opposes M1 polarization of microglia in the eye.

In the *rd1* model of RP, neither full-length CD200 nor its soluble ectodomain altered cone survival when overexpressed in the retina using an adeno-associated viral (AAV) vector (20). Nonetheless, augmenting CD200-CD200R signaling has been reported to be beneficial in other animal models of eye disease. For instance, intravitreal injection of DX109, a monoclonal antibody for CD200R with agonist activity, reduced the area of laser-induced CNV in mice compared to an isotype control (64). Intravitreal CD200Fc, another CD200R agonist, also decreased RGC apoptosis after optic nerve crush in rats (65), although its effect on RGCs subjected to elevated IOP has not been examined. In CD200 knockout mice, induction of EAU resulted in earlier and more severe disease activity as measured by histological scores and photoreceptor apoptosis (63). In contrast, EAU development in wild-type animals was suppressed following treatment with either systemic or intravitreal DX109 (66). Based on these studies, CD200R agonists such as DX109 and CD200Fc may be worth testing in patients with degenerative eye conditions. Another candidate is LY3454738, a CD200R agonist currently being evaluated in phase 1 trials for atopic dermatitis (NCT03750643) (67).

#### TGF-β

Transforming growth factor beta (TGF-β) is a pleiotropic cytokine with anti-inflammatory effects on microglia. When administered to microglial cultures, TGF-β inhibits the production of IL-1, IL-6, and TNF and downregulates major histocompatibility complex (MHC) class II, a marker for activated microglia (68, 69). Furthermore, silencing of TGF-β signaling specifically in microglia *via* deletion of TGF-β receptor 2 (TGFBR2) has been shown to cause activation of retinal microglia and death of photoreceptors and RGCs (70).

In mouse models of RP, ocular overexpression of TGF-β1, one of three TGF-β isoforms in mammals, saved cones from degeneration and preserved vision (21). Rescue of cones could be disrupted with either microglia depletion or blocking of TGF-β receptors, suggesting that treatment with TGF-β1 induced protection by microglia (21). TGF-β signaling in microglia may likewise be beneficial during AMD, as mice with microglia-specific ablation of TGFBR2 develop greater CNV after laser injury (70). However, the net effect of TGF-β in AMD eyes appears to be detrimental, since TGF-β inhibitors in both mice and rats have been found to reduce laser-induced CNV (71, 72). One possible reason for this discrepancy is that TGF-β receptors are present not only on microglia, but also on other ocular cell types, such as endothelial cells and the retinal pigment epithelium (RPE) (73). Using TGF-β to modulate microglia might therefore have unintended consequences if other parts of the eye respond in ways that have a negative impact. Reassuringly, AAV-mediated expression of TGF-β1 in wild-type mouse eyes produced no obvious adverse changes after a month (21). Nonetheless, the effects of TGF-β may be different depending on the context of ocular disease and in each case should be carefully evaluated.

#### TSPO Ligands

Translocator protein (TSPO) is a receptor on the outer mitochondrial membrane of microglia that becomes upregulated during their activation (74). When bound by its endogenous ligand in the eye, diazepam-binding inhibitor (DBI), TSPO suppresses features of activated microglia such as production of TNF and reactive oxygen species (74). In mice systemically treated with XBD173 (emapunil), a TSPO ligand with agonist activity, photoreceptor degeneration after acute exposure to bright light was reduced (75). However, in *rd10* mice, knockout of TSPO did not affect the rate of photoreceptor death (76), perhaps because degeneration in the *rd10* model is much more prolonged than that caused by bright light. TSPO signaling might therefore not be therapeutically relevant for genetic forms of retinal degeneration such as RP.

TSPO ligands have also been tested in the mouse laser injury model of AMD, although the results were not as straightforward. In wild-type mice, daily doses of XBD173 attenuated vascular leakage and CNV size (77), arguing that promotion of TSPO signaling may help in AMD. The same paper then found laser-induced CNV to improve with conditional deletion of TSPO in microglia (77), supporting the idea that inhibition of TSPO might instead be beneficial. Indeed, while knocking out TSPO has been shown to lower microglial expression of pro-inflammatory cytokines *in vitro* (78), knocking down TSPO reportedly results in the opposite (74, 79). These findings illustrate the complex and sometimes paradoxical nature of TSPO in regulating microglia and highlight a need for further studies of TSPO in eye disease.

#### IGF-1

Insulin-like growth factor 1 (IGF-1) is a pleiotropic polypeptide hormone that has been implicated in growth, development, and aging, among other biological processes. During neuroinflammation, IGF-1 is thought to enhance neuronal survival by reprogramming microglia to an M2 phenotype *via* the IGF-1R receptor (80). Consistent with this, intravitreal delivery of IGF-1 in *rd10* mice decreased photoreceptor apoptosis, and this effect was diminished in the presence of clodronate liposomes, which deplete microglia (81). IGF-1 has also been found to be neuroprotective for RGCs. In rats, intravitreal IGF-1 reduced RGC death following transection of the optic nerve (82). Likewise, intravitreal administration of insulin-like growth factor binding protein like-1 (IGFBPL1), which acts by binding to IGF-1, was able to slow visual decline in a microbead-induced mouse model of glaucoma (83).

Nonetheless, one challenge facing the use of IGF-1 as an ocular therapeutic is its potential to cause new pathology. Absence of IGF-1 in mice leads to progressive loss of ERG amplitudes and deterioration of retinal synapses (84). However, overexpression of IGF-1 similarly results in reduced ERG responses, as well as increased photoreceptor death and features of DR such as retinal microvascular damage (85, 86). Treating eyes with IGF-1 will thus require achieving intraocular levels sufficient to enable neuroprotection without triggering the various toxicities seen with excess IGF-1 signaling.

#### TUDCA

Tauroursodeoxycholic acid (TUDCA) is a conjugated bile acid that has shown promise in multiple models of eye disease. Specifically, systemic administration of TUDCA has been reported to slow retinal degeneration in both the *rd10* and P23H rodent models of RP (87, 88), preserve visual function in mice with STZ-induced DR (89), suppress CNV formation in rats after laser injury (90), and decrease photoreceptor death in rats after retinal detachment (91). Topical TUDCA was also found to delay RGC loss in rats following optic nerve crush (92), although the compound has not been tested in more physiological models of glaucoma. While use of TUDCA in P23H rats was associated with reduced activation of retinal microglia as supported by their downregulation of MHC class II (93), it is unknown how much this microglial reprogramming mediated TUDCA’s therapeutic effects. Furthermore, TUDCA has been reported to interact with numerous receptors both on and inside the cell (94), making its molecular mechanism uncertain. Regardless, given encouraging results with TUDCA in preclinical models and its documented tolerability in humans (95), trials employing it to treat ocular disorders should be considered.

#### Tamoxifen

Tamoxifen is a selective estrogen receptor modulator used to treat hormone receptor-positive breast cancer. In mice, tamoxifen is often also given to activate an inducible form of Cre recombinase that facilitates genetic changes in specific cell types. Unexpectedly, administration of tamoxifen-supplemented food in the *rd10* model of RP was found to slow photoreceptor degeneration compared to littermates receiving standard chow (96). Rescue of photoreceptors with tamoxifen was associated with decreased pro-inflammatory cytokine production by retinal microglia (96), implying that microglia were involved in the therapeutic mechanism. Although promising, the potential benefits of tamoxifen in RP will need to be reconciled with the drug’s well-documented ocular toxicities (97). These include crystalline deposits in the retina, foveal cavitations, and macular edema and are estimated to occur in up to 12% of patients who take the medication long-term (98).

#### GLP-1R Agonists

In 2017, it was reported that activated microglia can induce the formation of reactive astrocytes by secreting IL-1α, TNF, and complement component 1q (C1q) (99). These reactive astrocytes go on to promote neurodegeneration *via* production of an unknown toxin that kills a variety of CNS cell types, including RGCs (99). Recently, microglial induction of reactive astrocytes was observed in a mouse model of glaucoma created by injection of microbeads (100). RGC survival in these animals was improved by NLY01, a glucagon-like peptide 1 receptor (GLP-1R) agonist that has been shown to halt astrocyte transformation by suppressing microglial expression of IL-1α, TNF, and C1q (100, 101).

Initially developed to treat diabetes, GLP-1R agonists are a class of medications that augment glucose-dependent secretion of insulin to help with glycemic control and weight loss (102). However, GLP-1R agonists may additionally possess neuroprotective properties, which have been attributed to their actions on microglia and subsequently astrocytes (101, 103, 104). GLP-1R agonists have also been tested in mice with congenital diabetes, where they were found to decrease retinal apoptosis independent of their glucose-lowering effects (105). These studies suggest that beyond microglia, interventions targeting reactive astrocytes may likewise be relevant for degenerative eye diseases.

### Effect Blockade

A third strategy to prevent microglia-mediated degeneration in the eye is to directly target the downstream effects of these cells. These include the production of pro-inflammatory cytokines like IL-1, TNF, and C1q, as well as injurious behaviors such as the engulfment of stressed but viable neurons (106). Although not explored here, other actions of activated microglia such as the generation of reactive oxygen and nitrogen species also merit investigation in the context of ocular disease.

#### IL-1 Inhibitors

IL-1α and IL-1β (collectively referred to as IL-1) are potent pro-inflammatory cytokines that signal *via* the IL-1 receptor. In the eye, IL-1 is expressed by multiple cell types including microglia, which upregulate IL-1 in animal models of many degenerative eye disorders (21, 32, 100, 107). Elevated IL-1 levels have similarly been detected in the vitreous of patients with RP, AMD, and DR (108–110), as well as the subretinal fluid of patients with retinal detachment (111). Functionally, IL-1 is thought to indirectly cause neuronal damage by triggering glial production of neurotoxic molecules (99, 112, 113). Consistent with this, addition of IL-1β to explanted mouse retinas was sufficient to induce photoreceptor apoptosis and cone outer segment loss (114, 115).

In animal models, opposing IL-1 activity has generally been found to alleviate retinal degeneration. In *rd10* mice, intravitreal injections of anakinra, a recombinant IL-1 receptor antagonist (IL-1RA), reduced photoreceptor apoptosis compared to saline in contralateral eyes (32). Intravitreal anakinra likewise inhibited CNV in rats subjected to laser injury (116), while subcutaneous delivery of IL-1RA suppressed CNV in mice (117). After retinal detachment, IL-1β blockade *via* subretinal injections of a neutralizing antibody decreased photoreceptor death in mice (111). Mice deficient in the IL-1 receptor also exhibited fewer infiltrating cells following induction of EAU (107). Supporting this, lentiviral vector-mediated expression of IL-1RA in mouse eyes lessened the severity of uveitis elicited by intravitreal LPS (118).

Despite these encouraging results, the therapeutic potential of IL-1 blockade has yet to be realized for patients with degenerative eye diseases. In a small study of six subjects with active DR, systemic inhibition of IL-1β with canakinumab, a monoclonal antibody, had no effect on the area of neovascularization (119). Canakinumab was also used in a phase 1 study for treating exudative AMD that completed in 2007 without follow-up (NCT00503022), suggesting a likely unfavorable outcome. In other degenerative eye disorders, IL-1 inhibitors have still not been tested, even though there are now several of these compounds approved for clinical use. Repurposing these medications to block IL-1 activity in the eye might ameliorate ocular pathology and should be considered for future trials.

#### TNF Inhibitors

Tumor necrosis factor (TNF) is another pro-inflammatory cytokine upregulated by microglia during ocular disease. Originally named for its ability to lyse tumor cells, TNF is also a key driver of rheumatoid arthritis, inflammatory bowel disease, and multiple other inflammatory conditions. Structurally, TNF is synthesized as a transmembrane protein, which when cleaved releases soluble TNF (120). Both transmembrane and soluble TNF can signal *via* the two TNF receptors to activate pathways that trigger apoptosis and cytokine secretion (121). Moreover, autocrine TNF signaling can stimulate further TNF production by microglia and lead to glutamate release that exacerbates neurotoxicity (122, 123).

As with IL-1, inhibition of TNF appears to be broadly beneficial across animal models of eye disease. Indeed, TNF blockade with adalimumab, a monoclonal antibody, is clinically approved for the treatment of uveitis (124, 125). In the *rd10* model of RP, adalimumab slowed photoreceptor death when delivered either systemically or intravitreally (126, 127). Similarly, anti-TNF therapies have been reported to suppress laser-induced CNV in mice (128), rats (129), and non-human primates (130). In a rat model of glaucoma, RGC survival during ocular hypertension was improved with either XPro1595, which neutralizes soluble TNF, or etanercept, a TNF decoy receptor (131, 132). Furthermore, ablation of TNF signaling *via* knockout of TNF protected photoreceptors in mouse models of both DR and retinal detachment (133, 134).

In patients, TNF inhibitors have additionally exhibited promise for ocular diseases beyond uveitis. In a case series of three subjects with concurrent arthritis and AMD, treatment with systemic infliximab, a monoclonal antibody against TNF, led to CNV regression and better vision (135). In another series of three AMD patients, intravitreal administration of infliximab also restored sight (136). However, a slightly larger study of 13 participants found systemic infliximab to only stabilize visual acuity in AMD, rather than improve this measure (137). For DR, the effect of systemic infliximab on diabetic macular edema was previously examined in a small randomized controlled trial (138). Compared to the six placebo-treated eyes, which showed decreased vision after 16 weeks, the eight eyes receiving infliximab had significantly higher visual acuity that was improved from baseline (138). Blocking TNF thus appears to be a viable strategy to treat not only uveitis, but possibly also DR and AMD. Larger trials are warranted to fully test these ideas, as well as to evaluate the potential of TNF inhibitors in other degenerative eye conditions.

#### C1q Inhibitors

C1q was first discovered as part of C1, the protein complex that initiates the classical complement pathway (139). Since then, C1q has been shown to participate in many additional biological processes, ranging from the clearance of apoptotic cells to the pruning of excess synapses during development (140, 141). In both the brain and retina, C1q is primarily secreted by microglia and can become upregulated following microglial activation (21, 142). However, C1q levels are unchanged in some degenerative eye diseases such as DR (143), and interventions lowering ocular C1q *in vivo* have generated mixed results. For example, genetic deletion of C1q protected RGCs in the DBA/2J mouse model of glaucoma (144), but accelerated retinal degeneration in a mouse model of RP and had no impact on disease severity in EAU mice (145, 146). Inhibition of C1q by either small interfering RNA-mediated knockdown or knockout of the gene in mice also had no observable effect on laser-induced CNV (147, 148). Decreasing C1q may therefore be a suitable approach for glaucoma in particular, a notion supported by the elevation of C1q in both aqueous and vitreous samples from glaucoma patients (149, 150). This strategy was recently pursued in a phase 1b clinical trial using an intravitreal antigen-binding fragment to neutralize C1q in glaucomatous eyes (NCT04188015).

#### Cyclic RGD Peptides

Phagocytosis is a key component of microglial homeostasis that enables the clearance of pathogens, remodeling of synapses, and removal of dead and dying cells (151). However, phagocytosis can also become dysregulated following microglial activation, resulting in the engulfment and killing of viable cells (106). In 2015, Zhao et al. reported that microglia in the *rd10* model of RP phagocytose living rods, thereby accelerating retinal degeneration (32). Similarly, when incubated with fluorescent particles *ex vivo*, microglia from *rd1* retinas showed much higher phagocytotic activity than those from heterozygous mice (22).

The observation of phagocytic killing by microglia in a model of RP suggests that in some degenerative eye diseases, inhibitors of phagocytosis may be protective. Indeed, cyclic Arg-Gly-Asp (RGD)-containing peptides, which disrupt phagocytosis by blocking the vitronectin receptor on microglia (152, 153), have demonstrated efficacy in several animal models. In *rd10* mice, intravitreal delivery of cyclo(Arg-Gly-Asp-Phe-Val) led to greater preservation of rods and ERG amplitudes than an inactive analog injected into contralateral eyes (32). After laser injury, intravitreal administration of a cyclic RGD peptide in rats was also found to inhibit CNV progression (154). While the phagocytic activity of microglia in other ocular disorders has not been as thoroughly investigated, the potential of cyclic RGD peptides in these conditions should be explored.

#### CD47

Another molecule capable of inhibiting microglial phagocytosis is CD47, a transmembrane protein and well-established “don’t-eat-me signal.” In many cancers, upregulation of CD47 prevents the engulfment of tumor cells by binding to signal-regulatory protein alpha (SIRPα) on macrophages (155, 156). In the CNS, CD47 can analogously signal to microglia *via* SIRPα and has been reported to suppress their phagocytic activity during both development and disease (157, 158). When overexpressed on cones, CD47 protected against cone degeneration and vision loss in multiple mouse models of RP *via* a pathway that required SIRPα (22). However, rescue of cones was unperturbed by microglia depletion (22), suggesting that the effects of CD47-SIRPα signaling in these animals was likely carried out by non-microglial cells.

Interestingly, CD47 itself is also expressed on microglia, where it can interact with thrombospondin-1 (TSP1), a secreted matricellular protein (159). In a mouse model of AMD, TSP1 binding to endogenous CD47 helped resolve subretinal inflammation (159). TSP1-CD47 signaling therefore presents another potential avenue by which CD47 might ameliorate eye disease.

## Discussion

Despite their small numbers in the eye, microglia are increasingly being recognized as key contributors to ocular disease. As detailed in this review, strategies targeting microglia have likewise shown promise in animal models of eye disorders and may help patients retain their sight. In general, suppressing the pro-inflammatory activities of microglia appears to favor photoreceptor and RGC survival during retinal degeneration, although the extent of this varies across both interventions and diseases. While some compounds such as minocycline and TNF inhibitors have demonstrated efficacy in almost every animal model tested, others like C1q inhibitors may be more suitable for a single indication. Notably, depletion of nearly all microglia with CSF1R inhibitors was in multiple instances not helpful in combating pathology (19–24). One interpretation of this could be that microglia are not relevant in these conditions. However, this view is opposed by the fact that other interventions targeting microglia were able to alleviate degeneration in the same models. Instead, it is likely that even during disease, microglia still perform beneficial functions, which may be accentuated by approaches that reprogram microglia or block their injurious effects. This appears to be the case as well in neurodegenerative disorders of the brain like Alzheimer’s disease, where microglia have been reported to both promote and inhibit disease progression (160).

Nonetheless, there are many areas of microglial biology in which deeper investigation might lead to substantial improvements over existing therapies. First, the precision and durability of targeting microglia would benefit from the development of methods to genetically modify these cells. Unlike many cell types, microglia have so far not been amenable to transduction with viral vectors *in vivo* (161–163). Furthermore, gene delivery vehicles such as nanoparticles and AAV vectors often themselves elicit undesirable inflammatory responses from microglia (164–167). Finding a way to genetically alter microglia efficiently while minimizing inflammation thus represents a considerable challenge, but one that would enable highly tailored microglial reprogramming.

Efforts should also be taken to improve our understanding of microglia heterogeneity in the eye. In the brain, single-cell studies have revealed the existence of diverse microglia subtypes, including some specific to neurological disorders (168). Analogously, there are likely different populations and states of microglia in the eye with distinct functions during ocular disease. Supporting this, recent papers have described subsets of retinal microglia in mice associated with oxygen-induced retinopathy or light-induced photoreceptor degeneration (169, 170). It is possible that future characterization of microglia diversity in human eyes may allow for the identification of therapeutic targets unique to pathologic microglia.

Lastly, as microglia are present throughout the CNS, it would be worthwhile to optimize delivery strategies that act upon microglia in the retina, but not the brain or spinal cord. Because the eye is an enclosed compartment, this can be achieved if compounds are injected into the vitreous, subretinal, or suprachoroidal space. However, these routes are less convenient than systemic ones such as oral administration, especially if a treatment warrants repeat dosing. Topical instillation of therapies as eye drops would offer an approach that is both local and non-invasive if medications delivered this way could reach the retina. While adequate penetration of topical molecules to the posterior segment remains difficult (171), successful implementation of this route would make it much easier to target microglia in the eye.

## Author Contributions

All authors listed have made a substantial, direct, and intellectual contribution to the work and approved it for publication.

## Funding

This work was supported by a National Eye Institute T32 Postdoctoral Fellowship (SW) and the Howard Hughes Medical Institute (CC).

## Conflict of Interest

The authors declare that the research was conducted in the absence of any commercial or financial relationships that could be construed as a potential conflict of interest.

## Publisher’s Note

All claims expressed in this article are solely those of the authors and do not necessarily represent those of their affiliated organizations, or those of the publisher, the editors and the reviewers. Any product that may be evaluated in this article, or claim that may be made by its manufacturer, is not guaranteed or endorsed by the publisher.
